# Covalent Capture of Nanoparticle-Stabilized Oil Droplets via Acetal Chemistry Using a Hydrophilic Polymer Brush

**DOI:** 10.1021/acs.langmuir.4c03897

**Published:** 2024-12-06

**Authors:** Saul J. Hunter, Evelin Csányi, Joshua J. S. Tyler, Mark A. Newell, Matthew A. H. Farmer, Camery Ma, George Sanderson, Graham J. Leggett, Edwin C. Johnson, Steven P. Armes

**Affiliations:** † School of Chemistry, Joseph Banks Laboratories, University of Lincoln, Brayford Pool, Lincoln, Lincolnshire LN6 7TS, U.K.; ‡ Dainton Building, Department of Chemistry, The University of Sheffield, Brook Hill, Sheffield, South Yorkshire S3 7HF, U.K.; § GEO Specialty Chemicals, Hythe, Southampton, Hampshire SO45 3ZG, U.K.

## Abstract

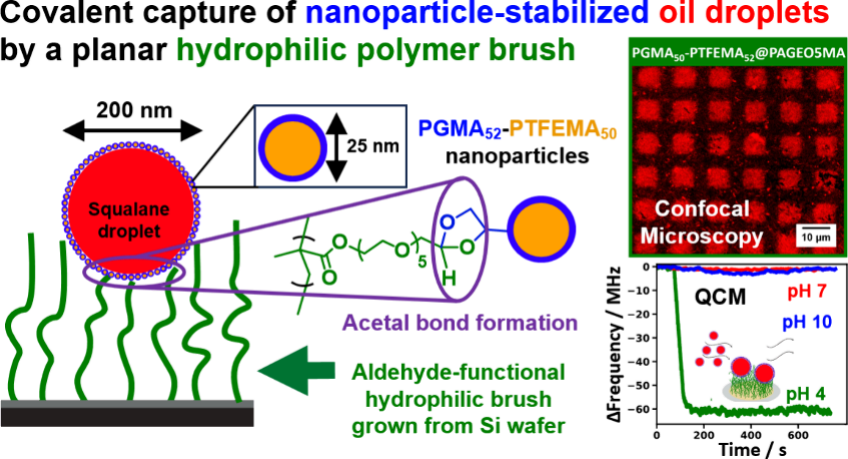

We report the capture of nanosized oil droplets using a hydrophilic aldehyde-functional polymer brush. The brush was obtained via aqueous ARGET ATRP of a *cis*-diol-functional methacrylic monomer from a planar silicon wafer. This precursor was then selectively oxidized using an aqueous solution of NaIO_4_ to introduce aldehyde groups. The oil droplets were prepared by using excess sterically stabilized diblock copolymer nanoparticles to prepare a relatively coarse squalane-in-water Pickering emulsion (mean droplet diameter = 20 μm). This precursor was then further processed via high-pressure microfluidization to produce ∼200 nm squalane droplets. We demonstrate that adsorption of these nanosized oil droplets involves acetal bond formation between the *cis*-diol groups located on the steric stabilizer chains and the aldehyde groups on the brush. This interaction occurs under relatively mild conditions and can be tuned by adjusting the solution pH. Hence this is a useful model system for understanding oil droplet interactions with soft surfaces.

## Introduction

A brush comprises polymer chains that are tethered to a surface by at least one chain end.^1−3^ Brushes can be prepared at planar or colloidal substrates using either a “grafting to”^4−7^ or a “grafting from” approach,^8−14^ with the latter approach usually providing a higher surface density of brush chains.^2,8,12,15^ In principle, brushes provide well-defined surface layers to examine soft matter interactions at the nanoscale.^16−24^

There are many reports of inorganic nanoparticle-decorated polymer brushes grown from planar substrates.^25^ Examples include gold nanoparticles within hydrophobic polystyrene or hydrophilic cationic methacrylic brushes,^26,27^ gold nanoparticles within pH-responsive poly(2-vinylpyridine) brushes,^28,29^ silver nanoparticles within zwitterionic brushes for antibacterial surfaces,^30^ or within poly(2-(dimethylamino)ethyl methacrylate) brushes as a surface-enhanced Raman scattering (SERS) sensor,^31^ gold nanoparticles within poly(oligo(ethylene glycol) methacrylate) brushes as a lead ion sensor,^32^ and quantum dot nanoparticles with poly(acrylic acid) brushes.^33^

In some cases, nanoparticle adsorption may simply involve nonspecific electrostatic attraction or van der Waals interactions. However, there are various reports of chemically reactive polymer brushes in the literature, including poly(2-hydroxyethyl methacrylate),^34,35^ poly(glycidyl methacrylate),^36^ poly(2-(dimethylamino)ethyl methacrylate),^37^ poly(2-(*tert*-butylamino)ethyl methacrylate)^38^ and poly(cysteine methacrylate).^39^

We have exploited dynamic covalent chemistry to demonstrate the pH-modulated adsorption of a series of sterically stabilized diblock copolymer nanoparticles onto model polymer brushes.^40−43^ Originally, our approach utilized phenylboronic acid binding,^42^ but more recently we have explored Schiff base chemistry^41,44^ and acetal bond formation.^40,43^

**Scheme 1:**
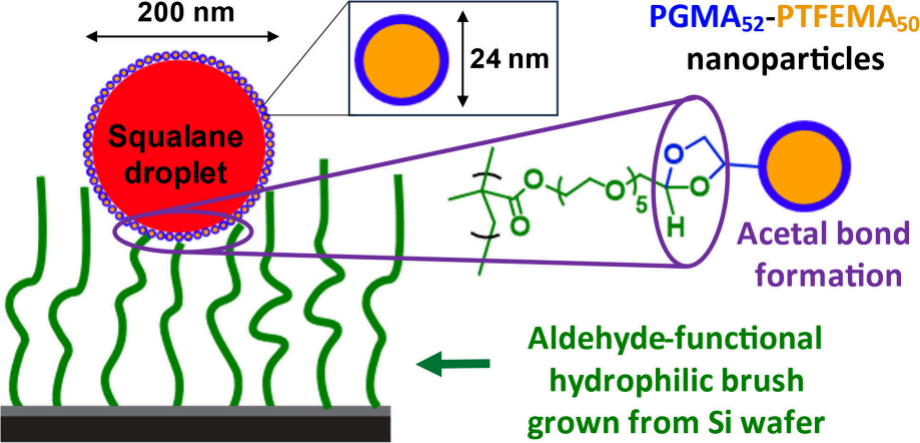
Covalent Attachment of Hydrophobic Nanoparticle-Stabilized Oil Droplets to a Hydrophilic Aldehyde-Functional Brush An oil-in-water Pickering nanoemulsion was prepared via high-pressure microfluidization using squalane and PGMA_50_-PTFEMA_52_ nanoparticles. The aldehyde-functional brush was obtained by growing a *cis*-diol-functional brush from a planar silicon wafer followed by NaIO_4_ oxidation in aqueous solution.

Herein we report the capture of *hydrophobic* oil droplets of ∼200 nm diameter using a *hydrophilic* aldehyde-functional brush grown from a planar substrate (see Scheme 1). The oil droplets are stabilized using 24 nm diameter sterically stabilized diblock copolymer nanoparticles, which are conveniently prepared by polymerization-induced self-assembly (PISA).^45−49^ The steric stabilizer chains contain pendent *cis*-diol groups, which react with pendent aldehyde groups located on the brush chains to form acetal bonds. Such covalent attachment can be modulated simply by adjusting the solution pH. The resulting oil droplet-decorated brush layer is characterized by confocal and fluorescence microscopy studies and quartz crystal microbalance (QCM) measurements.

## Materials and Methods

Full synthesis and characterization details are provided in the experimental section within the Supporting Information.

## Results and Discussion

**Figure 1:**
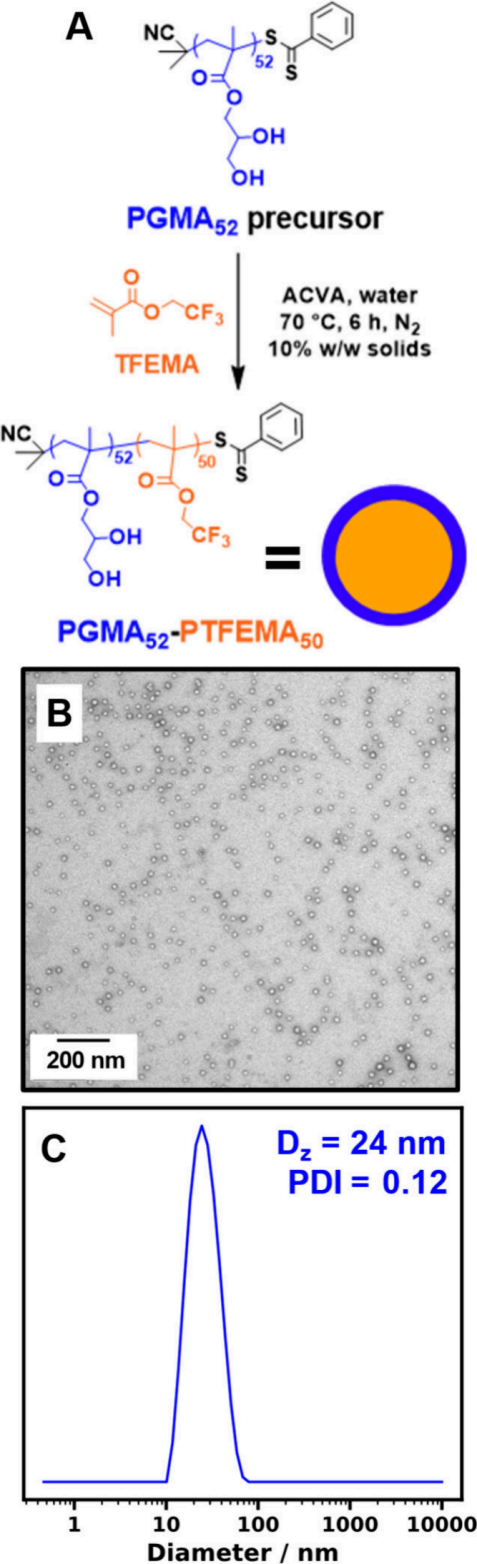
(A) Synthetic route for the preparation of *cis*-diol-functional PGMA_52_-PTFEMA_50_ nanoparticles via RAFT aqueous emulsion polymerization of TFEMA at 70 °C. (B) Representative TEM image (uranyl formate stain) and (C) DLS size distribution obtained for PGMA_52_-PTFEMA_50_ nanoparticles.

The sterically stabilized PGMA–PTFEMA nanoparticles used in this work were prepared by reversible addition–fragmentation chain transfer (RAFT) aqueous emulsion polymerization of 2,2,2-trifluoroethyl methacrylate (TFEMA) using a PGMA_52_ precursor (Figures 1A and S1).^47^

A representative transmission electron microscopy (TEM) image of such nanoparticles is shown in Figure 1B, while an intensity-average particle size distribution obtained by dynamic light scattering (DLS) is provided in Figure 1C. The latter technique indicates a z-average diameter of 24 nm and a DLS polydispersity of 0.12. Clearly, these nanoparticles possess a well-defined spherical morphology and a relatively narrow unimodal particle size distribution.

The steric stabilizer block used for the synthesis of these nanoparticles is poly(glycerol monomethacrylate) (PGMA), which has one pendent *cis*-diol group per repeat unit. Assuming an aggregation number of 320 (estimated from TEM analysis assuming a PTFEMA density of 1.47 g cm^–3^)^49^ and a mean degree of polymerization of 52 for the PGMA chains, we calculate that each nanoparticle contains approximately 16,600 *cis*-diol groups. Such nanoparticles have been previously used to prepare various types of oil-in-water Pickering nanoemulsions.^47,50−54^

In a recent study, closely related nanoparticles were selectively oxidized to convert the pendent *cis*-diol groups into aldehyde groups. The resulting nanoparticles were used to prepare the corresponding Pickering nanoemulsions, which exhibited strong mucoadhesion when exposed to sheep nasal mucosa.^46^ In this case, nanoparticle adsorption was mediated by imine bond formation (Schiff base chemistry) between the surface aldehyde groups and the primary amine groups associated with proteins within the biological tissue.

Previously, we reported the synthesis of aldehyde-functional polymer brushes.^41,44^ This involved surface polymerization of a *cis*-diol-functional methacrylic monomer (denoted GEO5MA) from a planar silicon wafer, followed by selective oxidation of the resulting PGEO5MA brush to produce the desired aldehyde-functionalized PAGEO5MA brush. The chemical oxidation conditions were optimized to minimize surface degrafting and the resulting dense brushes proved to be reactive toward a model globular protein (BSA). In contrast, the precursor *cis*-diol-functional brush exhibited antibiofouling properties.^44^ More recently, we examined the pH-modulated adsorption of either spherical nanoparticles of varying size^40^ or enzyme-loaded diblock copolymer vesicles^43^ onto such aldehyde-functional brushes. In this case, the nanoparticle–brush interaction is mediated by acetal bond formation between the *cis*-diol functional nanoparticles and the aldehyde-functional brush chains. Moreover, such covalent bond formation could be modulated by varying the solution pH: strong nanoparticle adsorption occurred at pH 4, whereas little or no adsorption was observed at pH 10.^40,55,56^

**Figure 2:**
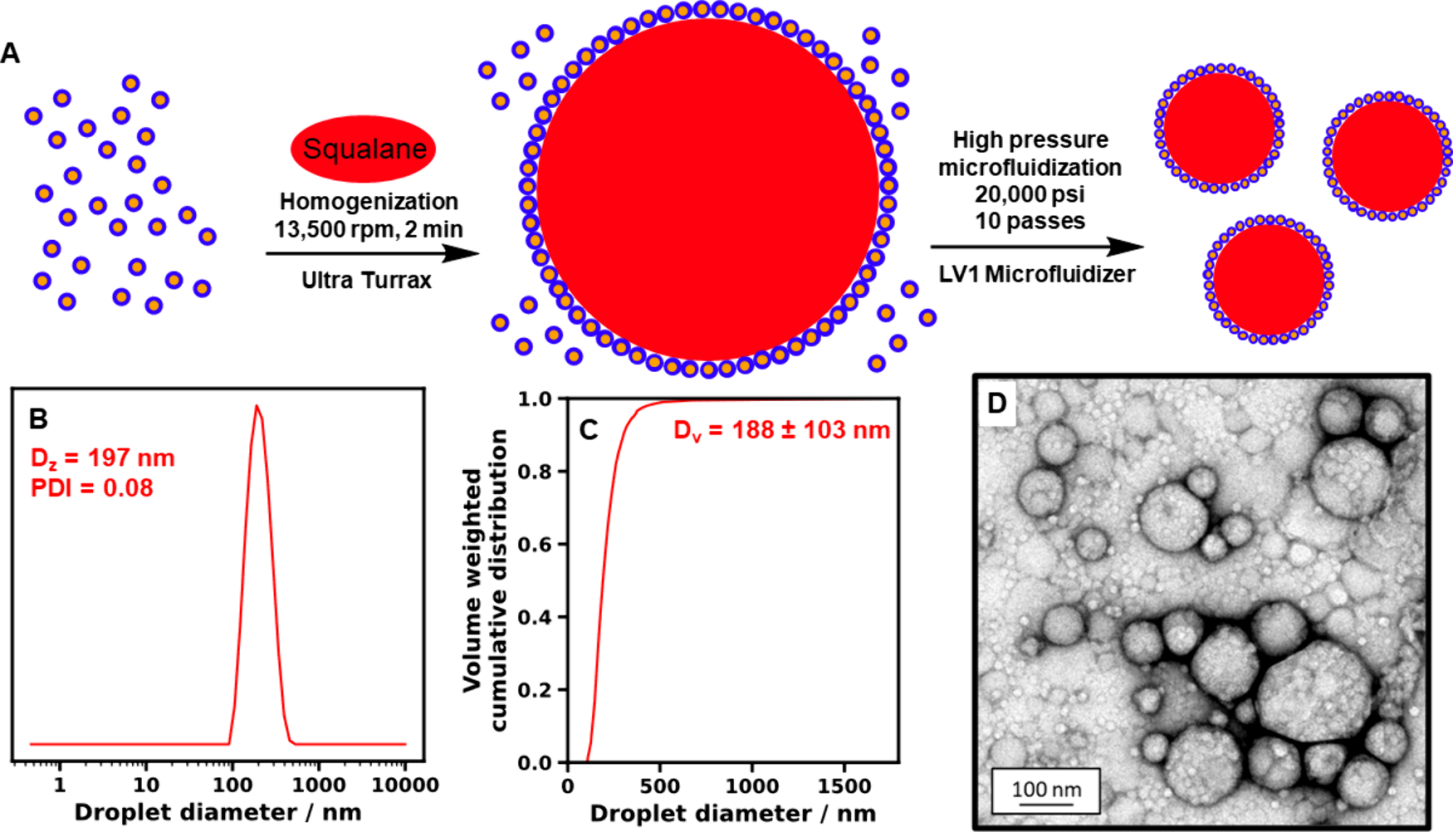
(A) Schematic representation for the formation of O/W Pickering nanoemulsions using PGMA_52_–PTFEMA_50_ nanoparticles as a Pickering emulsifier. First, high-shear homogenization of 2.5–10% w/w aqueous dispersions of such nanoparticles with 20% v/v squalane resulted in the formation of a Pickering macroemulsion with a mean droplet diameter of 20 μm. Then this precursor macroemulsion was passed ten times through a high-pressure microfluidizer at 20,000 psi to produce the desired O/W Pickering nanoemulsion with a mean droplet diameter of approximately 200 nm. (B) DLS size distribution, (C) cumulative size distribution via analytical centrifugation (LUMiSizer instrument), and (D) representative TEM image for the O/W Pickering nanoemulsion obtained using this protocol.

In the present study, we examine whether the same aldehyde-functional brush system can be used to capture nanosized oil droplets. We identified squalane as a suitable oil because its very low aqueous solubility minimizes the problem of Ostwald ripening over time, which is well-documented for such Pickering nanoemulsions.^47^ First, a relatively coarse Pickering emulsion was prepared via high-shear homogenization using a large excess of PGMA_52_-PTFEMA_50_ nanoparticles (Figure 2A). Optical microscopy and laser diffraction studies indicated droplet diameters ranging from 10 to 50 μm. This precursor was then further processed via high-pressure microfluidization (see the Supporting Information for further details) to produce the final Pickering nanoemulsion, as described previously.^47,50−54^ Subsequent DLS characterization indicated a mean z-average droplet diameter of approximately 200 nm (Figure 2B), which is consistent with analytical centrifugation studies using a LUMiSizer instrument (see Figure 2C). A representative TEM image obtained for this freshly prepared nanoemulsion is shown in Figure 2D. Both the squalane droplet phase and the continuous phase evaporate under ultrahigh vacuum conditions, leaving only the nanoparticles that were adsorbed at the surface of the oil droplets. These nanoparticles form spherical superstructures that are comparable in size to the mean DLS diameter of the original oil droplets. Moreover, close inspection revealed the presence of the individual nanoparticles, which is consistent with TEM observations made in our prior study.^47^ Thus the nanoparticles survive the high-pressure microfluidization processing conditions and the Pickering nature of the original nanoemulsion is confirmed.

**Figure 3:**
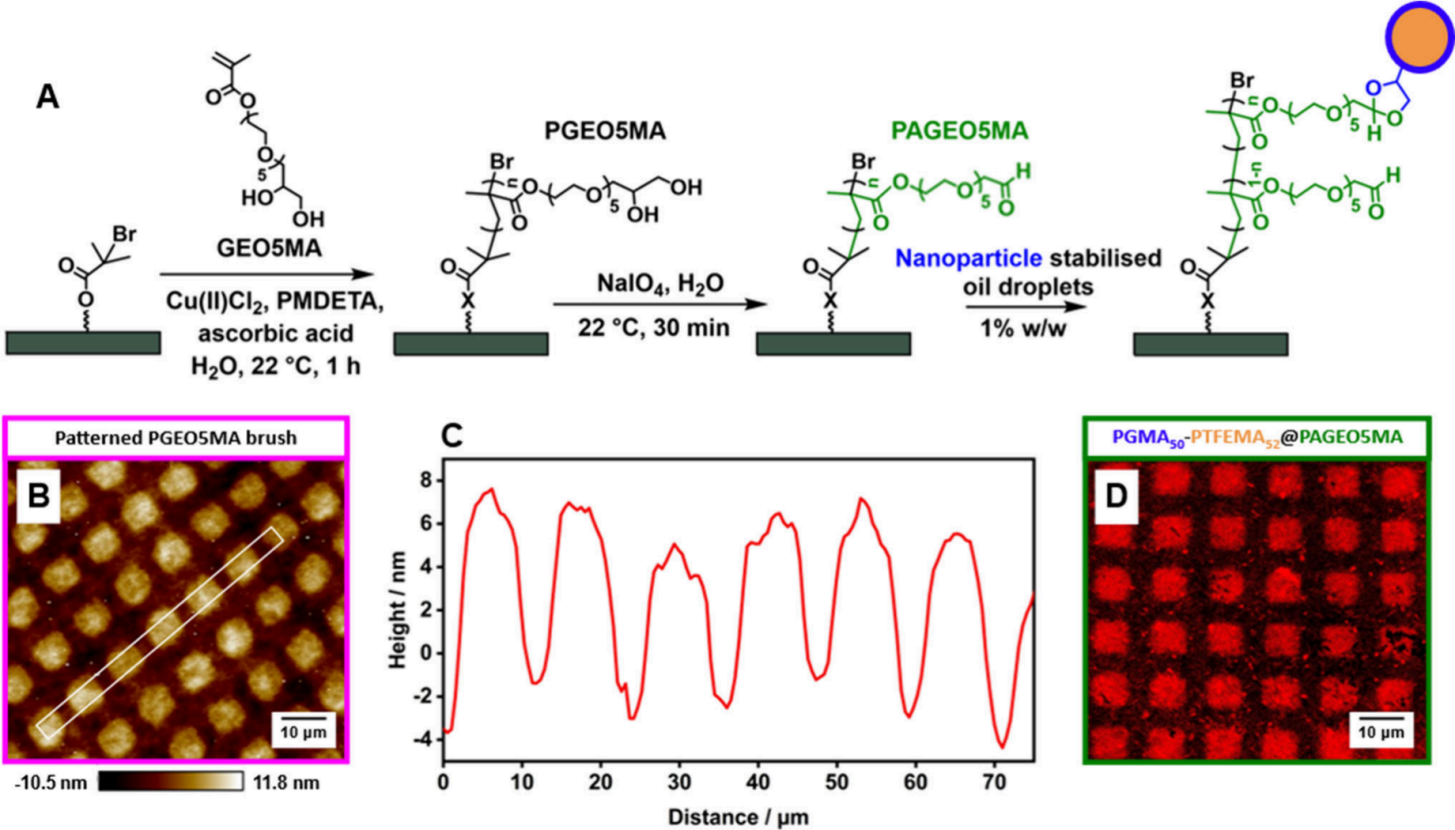
(A) (i) Preparation of a *cis*-diol functional PGEO5MA precursor brush via ARGET ATRP from a initiator-functionalized planar silicon wafer, (ii) selective oxidation to produce the corresponding aldehyde-functional PAGEO5MA brush, and (iii) its subsequent exposure to a squalane-in-water Pickering nanoemulsion prepared using PGMA_52_-PTFEMA_50_ nanoparticles (with Nile Red dye dissolved in the oil phase prior to nanoemulsion formation). (B) AFM image of a patterned PGEO5MA brush with (C) corresponding brush height profile, as indicated through the line profile [see white box shown in (B)]. (D) Confocal fluorescence micrograph recorded for a *surface-patterned* PAGEO5MA brush after exposure to the dilute nanoemulsion [N.B. This image was recorded for the water-swollen brush to minimize droplet evaporation].

The preparation of the hydrophilic aldehyde-functionalized PAGEO5MA brush is summarized in Figure 3A. Briefly, *cis*-diol-functionalized PGEO5MA precursor brushes were prepared by performing ARGET ATRP from surface-patterned initiator-functionalized silicon wafers. Patterned surfaces were prepared via UV deprotection of nitrophenyl-protected APTES (NPPOC-ATPES) via irradiation using an appropriate mask (Scheme S1 and Figure S2).^57^ The mean dimensions of each square were 10 × 10 μm^2^. The resulting square-patterned PGEO5MA brush was characterized by atomic force microscopy (AFM), see Figure 3B. The corresponding height profile is shown in Figure 3C and indicates a somewhat thinner patterned brush (dry brush thickness ∼15 nm) compared to the equivalent nonpatterned brush.^44^ This discrepancy is attributed to the lower density of surface initiator sites for the patterned brush. The pendent *cis*-diol groups were then selectively oxidized under mild conditions using NaIO_4_, as reported previously.^44^ Prior XPS studies indicated that this protocol produces a PAGEO5MA brush with essentially 100% aldehyde functionality.^44^ For this chemical derivatization, the reaction time was limited to 30 min to minimize brush degradation.^44^

Given the hydrophilic character of the brush chains and the hydrophobic nature of the oil droplets, we felt that successful droplet adsorption was not necessarily guaranteed and arguably counterintuitive. Nevertheless, oil droplet capture by PAGEO5MA brush chains was achieved at pH 4. This is illustrated in Figure 3D, which shows a confocal microscopy image recorded for a square-patterned PAGEO5MA brush grown from a wafer after its immersion into a dilute (1.0% w/w) Pickering nanoemulsion for 16 h at 20 °C. Nile Red dye was dissolved in squalane prior to nanoemulsion preparation to aid visualization of the oil droplets. Clearly, there is some degree of nonspecific adsorption of oil droplets to the underlying silicon wafer, which results in a weakly fluorescent background signal. However, a much stronger signal is observed for the patterned brush regions (see Figure 3D). Hence this experiment confirms that chemical adsorption of hydrophobic oil droplets onto a hydrophilic polymer brush can be achieved under mild reaction conditions. Moreover, there is no evidence for adsorption-induced droplet coalescence. In contrast, Rannard and co-workers reported that significant coalescence occurred during the adsorption of relatively large (∼10–20 μm diameter) mucoadhesive oil droplets onto a Carbopol-based synthetic mucous surface.^58^ This difference is attributed to the much finer oil droplets used in the present study. It is perhaps worth emphasizing that the “Pickering” nature of the oil droplets is an essential aspect of our strategy for ensuring the chemical adsorption of nanosized oil droplets: each nanoparticle contains many *cis*-diol surface groups, which facilitates strong interaction between the oil droplets and the aldehyde-functional brush chains.

Recently, we used a QCM instrument to demonstrate that the adsorption of *cis*-diol-functionalized diblock copolymer nanoparticles onto aldehyde-functionalized brushes involves acetal bond formation.^40^ This well-known chemistry is known to be favored at low pH, which is consistent with the strong nanoparticle adsorption observed under such conditions.^40^ In striking contrast, only minimal adsorption occurred at either pH 7 or pH 10. Moreover, higher adsorbed amounts were observed at higher temperature, which ruled out a purely physical interaction.^40^

**Figure 4:**
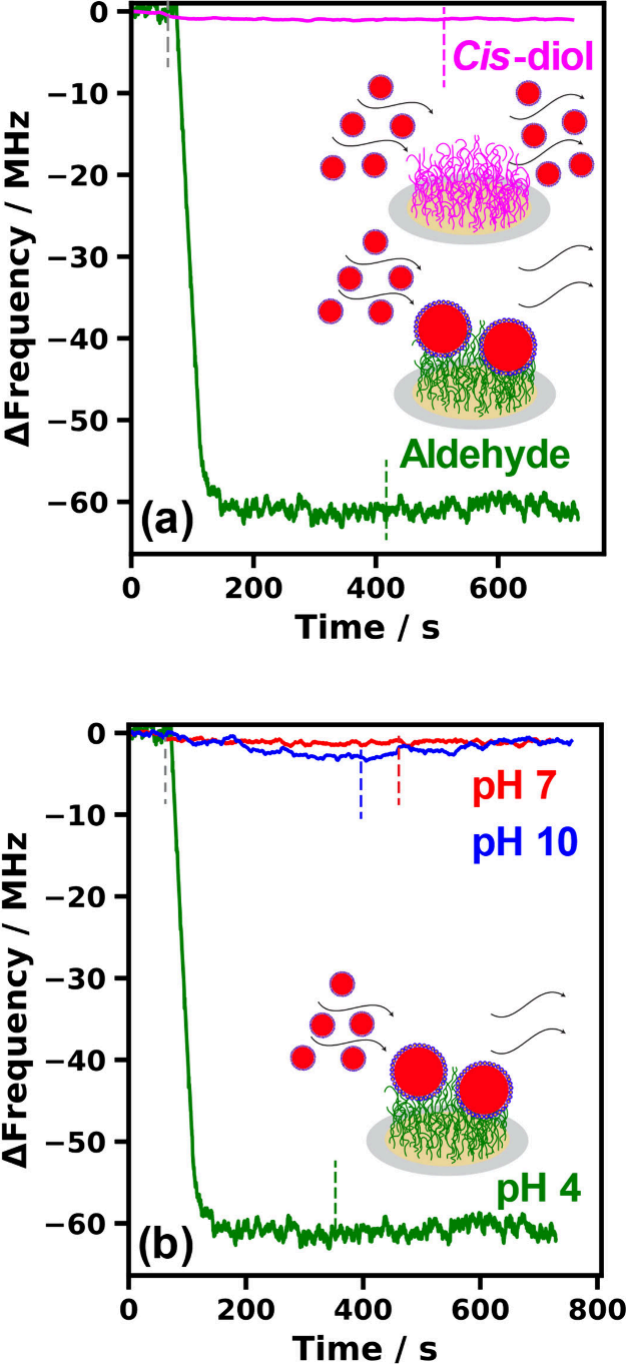
Change in frequency of the third overtone, Δ*f*_3_, over time at 25 °C for QCM analysis of silica sensors coated with (a) a 41 nm aldehyde-functional PAGEO5MA brush and the corresponding *cis*-diol-functional PGEO5MA precursor brush on exposure to a 1% w/w squalane-in-water Pickering nanoemulsion at pH 4 or (b) a 41 nm aldehyde-functional PAGEO5MA brush on exposure to a 1% w/w squalane-in-water Pickering nanoemulsion at pH 4, 7, or 10. In each case, the vertical dashed line indicates the time at which flow was switched to a purely aqueous solution (i.e., attempted wash-off of any weakly adhering adsorbed material).

Accordingly, adsorption of the oil droplets onto the brush layers was studied using the same QCM instrument. First, a direct comparison was made between the oil droplet capture performance of an aldehyde-functionalized PAGEO5MA brush and the corresponding *cis*-diol-functionalized PGEO5MA brush at pH 4 (see Figure 4a). As expected, almost no oil droplet adsorption was obtained in the latter case, whereas strong adsorption was observed in the former case. In a second series of experiments, the solution pH was adjusted when exposing the same aldehyde-functionalized PAGEO5MA brush to the dilute Pickering nanoemulsion. Strong oil droplet adsorption was observed at pH 4, whereas minimal adsorption occurred at either pH 7 or pH 10 (see Figure 4b). This is consistent with our recent study of the adsorption of *cis*-diol-functionalized diblock copolymer nanoparticles of varying size onto an aldehyde-functionalized brush layer.^40^ Moreover, such observations provide indirect evidence for chemical adsorption via acetal bond formation between the *cis*-diol groups present at the surface of the oil droplets and the aldehyde groups on the brush chains. The QCM data summarized in Figure 4 also confirm that there is essentially no physical interaction (e.g., attractive van der Waals forces) between the oil droplets and the brush chains.

## Conclusions

A suitably reactive hydrophilic polymer brush can be used to capture nanoparticle-stabilized oil droplets. This counterintuitive result is achieved via acetal bond formation between the aldehyde groups located on the brush chains and the *cis*-diol groups expressed at the surface of the nanoparticles surrounding the oil droplets. Such chemical adsorption occurs in aqueous media under relatively mild conditions and the droplet-brush interaction can be modulated by varying the solution pH. This well-defined model system offers an interesting opportunity to understand the interaction between nanosized oil droplets and well-defined soft surfaces.

## Supporting Information Available

Full experimental details for all syntheses and characterization methods, including nanoparticle synthesis, nanoemulsion preparation, preparation of initiator-functionalized silicon wafers and glass coverslips, synthesis of the NPPOC-APTES reagent, PGEO5MA brush syntheses and their selective oxidation, oil droplet adsorption, plus further ^1^H NMR spectroscopy, GPC, DLS, TEM, analytical centrifugation, atomic force microscopy, confocal microscopy, ellipsometry, and QCM data (PDF)
