# Supplementary material for: Covalent Capture of Nanoparticle-Stabilized Oil Droplets via Acetal Chemistry Using a Hydrophilic Polymer Brush
Source: Langmuir. 2024 Dec 6;40(50):26735–41. doi: 10.1021/acs.langmuir.4c03897 (PMC11656699; doi:10.1021/acs.langmuir.4c03897)
Supplement: Supplementary file 1 — la4c03897_si_001.pdf [file la4c03897_si_001.pdf]

Supporting Information for:

Covalent capture of nanoparticle-stabilized oil droplets via acetal chemistry using a hydrophilic polymer brush

Saul J. Hunter,[a] Evelin Csányi,[b] Joshua J. S. Tyler,[b] Mark A. Newell,[b] Matthew A. H. Farmer,[b] Camery Ma,[b] George Sanderson,[c] Graham J. Leggett,[b] Edwin C. Johnson[b]* and Steven P. Armes[b]*

a. School of Chemistry, Joseph Banks Laboratories, University of Lincoln, Brayford Pool, Lincoln, LN6 7TS, Lincolnshire, UK.

b. Dainton Building, Department of Chemistry, The University of Sheffield, Brook Hill, Sheffield, South Yorkshire, S3 7HF, UK.

c. GEO Specialty Chemicals, Hythe, Southampton, Hampshire SO45 3ZG, UK.

Experimental

Materials

All chemicals were used without further purification, unless otherwise stated. *N,N,N',N",N"*-pentamethyldiethylenetriamine (PMDETA, >99.0%), and sodium hydroxide were purchased from Fisher Scientific, UK. Dichloromethane (DCM, 99.8%), sodium periodate ($NaIO_4$, ≥99.8%), (30aminopropyl)triethoxysilane (APTES, >99%), triethylamine (TEA, 99%), 2-bromoisobutyryl bromide (BiBB, >99%), copper(II) chloride ($CuCl_2$, 99.99%), and ascorbic acid (AscAc, >98%), were purchased from Sigma-Aldrich Ltd (UK). GEO5MA monomer was prepared according to our recently reported experimental protocol.[1] Deionized water (pH 6.8) was obtained using an Elga Elgastat Oprion 3A water purification system. Native oxide-coated silicon wafers were purchased from Pi-KEM, Tamworth, UK. Fluorescein 5(6)-isothiocyanate (>90%) and sodium phosphate dibasic salt (>99.5%) were purchased from Sigma-Aldrich and used as received. Glycerol monomethacrylate GMA; 99.8% purity) was obtained from GEO Specialty Chemicals (Hythe, U.K.). 2,2,2-Trifluoroethyl methacrylate (TFEMA, 99%), 4,4′-azobis(4-cyanopentanoic acid) (ACVA, ≥98.0%), n-dodecane (>99%), squalane (96%), and were all purchased from Sigma-Aldrich (U.K.). 2-Cyano-2-propyl dithiobenzoate (CPDB, >97%) was purchased from Strem Chemicals Ltd. (Cambridge, U.K.). d_6-Acetone and d_4-methanol (CD_3OD) were purchased from Goss Scientific Instruments Ltd. (Cheshire, U.K.). Benzyltrimethylammonium hydroxide solution (40 wt.% in methanol), 1-ethyl-2-nitrobenzene (96%), 3-(triethoxysilyl)propyl isocyanate (95%) and diethyl ether (≥99.8%, contains BHT as inhibitor, puriss. p.a., ACS reagent, reag. ISO, reag. Ph. Eur.) were purchased from Sigma-Aldrich, UK. Paraformaldehyde (97%) was purchased from Alfa Aesar, UK. Hydrochloric acid (35%) and petroleum spirit 40-60 °C (max. 0.01% aromatic hydrocarbons) were purchased from VWR International, UK. Ethyl acetate (99.5%, HPLC grade), magnesium sulphate (laboratory reagent grade), phosphate buffered saline tablets,

hydrogen peroxide (100 volumes, > 30% w/v), sulfuric acid (S.G. 1.83, 95%, laboratory reagent grade), ethanol (absolute, 99.8%, HPLC grade) and toluene (99.8%, HPLC grade) were purchased from Fisher Scientific, UK.

Synthesis protocols

Synthesis of a $PGMA_{52}$ precursor via RAFT solution polymerization in ethanol

GMA (30.1 g, 0.186 mol), CPDB (0.589 g, 2.66 mmol; target DP = 70), ACVA (0.149 g, 0.532 mmol; CPDB/ACVA molar ratio = 5.0), and anhydrous ethanol (45.9 g) were weighed into a 100 mL round-bottom flask. The resulting solution was deoxygenated by purging with a stream of nitrogen gas for 30 min at 20 °C before the flask was immersed in an oil bath at 70 °C for 165 min with continuous stirring. The polymerization was quenched by removing the flask from the oil bath and exposure of the reaction mixture to air while cooling the flask to 20 °C. A GMA conversion of 72% was determined by ^{1}H NMR spectroscopy by comparing the integrated monomer vinyl signals at 5.7 and 6.2 ppm with the five pendent PGMA proton signals at 3.7–4.3 ppm. The crude polymer was purified by precipitation into a ten-fold excess of DCM (three times) and then freeze-dried from water. A mean DP of 52 was determined via end-group analysis using ^{1}H NMR spectroscopy (d_4-methanol) by comparing the integrated peaks of the aromatic protons assigned to the dithiobenzoate chain ends at 7.4–7.9 ppm to the five pendent GMA protons at 3.7–4.3 ppm. DMF GPC analysis (using a UV detector set at 298 nm) indicated an M_n of 13 100 g mol^{-1} and an M_w/M_n of 1.21.

Synthesis of $PGMA_{52}$–$PTFEMA_{50}$ diblock copolymer nanoparticles via RAFT aqueous emulsion polymerization of TFEMA

A $PGMA_{52}$ precursor (2.50 g, 0.304 mmol), ACVA (0.0167 g, 0.0608 mmol; $PGMA_{52}$/ACVA molar ratio = 5.0), and deionized water (45.6 g) were added to a 100 mL round-bottom flask. The resulting aqueous solution was then degassed with a stream of nitrogen gas for 30 min

at 20 °C. TFEMA (2.16 mL, 0.0152 mol; target DP = 50 at 10% w/w solids) was degassed separately using an ice bath to minimize evaporation and then injected into the round-bottom flask prior to its immersion in an oil bath at 70 °C for 6 h. The ensuing TFEMA polymerization was quenched by exposing the reaction mixture to air while cooling the flask to 20°C. ^{19}F NMR spectroscopy analysis in d_6-acetone indicated more than 99% TFEMA conversion (the integrated TFEMA monomer signal at –74.6 ppm was compared to the integrated PTFEMA signal at –73.9 ppm). DMF GPC analysis (using a UV detector set at 298 nm) indicated an M_n of 22 000 g mol^{-1} and an M_w/M_n of 1.19. A ^{1}H NMR spectrum of this PGMA$_{52}$–PTFEMA$_{50}$ diblock copolymer is shown in **Figure S1**.

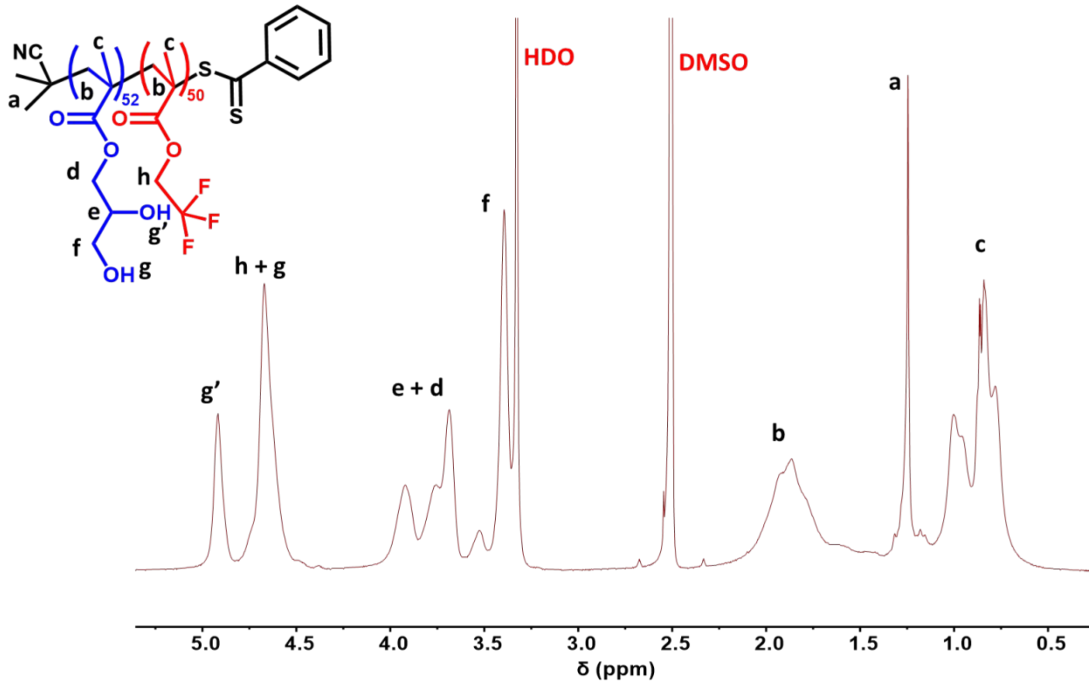

Figure S1. ^{1}H NMR spectrum (d$_6$-DMSO) recorded for the PGMA$_{52}$–PTFEMA$_{50}$ diblock copolymer showing the characteristic proton signals for each block.

Preparation of Pickering macroemulsion by high-shear homogenization

An aqueous dispersion of PGMA$_{52}$–PTFEMA$_{50}$ nanoparticles (2.40 mL, 8.0% w/w) was added to a 14 mL glass vial and homogenized with squalane (0.60 mL) at 13 500 rpm for 2 min at 20 °C using an IKA Ultra-Turrax T-18 homogenizer with a 10 mm dispersing tool.

Preparation of Pickering nanoemulsion by high-pressure microfluidization

The above Pickering macroemulsion (3.0 mL) was further processed with the aid of an LV1 microfluidizer (Microfluidics). The applied pressure was 20 000 psi and each macroemulsion was passed through the LV1 ten times to produce a Pickering nanoemulsion.

Surface functionalization of planar silicon wafer with ATRP initiator groups

ATRP initiator-functionalized silicon wafers were prepared using a previously reported protocol.[2,3] Pieces of wafer (~ 1 x 1 cm^2) were subjected to UV-ozone cleaning for 30 min using a Bioforce Nanosciences ProCleaner (BioForce Nanosciences, USA) prior to immersion in 0.5 M NaOH and copious rinsing with deionized water. APTES was vapor-deposited onto clean wafers for 30 min at 22 °C before annealing at 110 °C for 30 min. These APTES-functionalized wafers were then immersed in a solution of 2-bromoisobutyryl bromide (BiBB) in dichloromethane in the presence of triethylamine (conditions: DCM/TEA/BiBB = 400:1:1 by volume) and the surface amidation reaction was allowed to proceed for 1 h at 22 °C. Finally, the resulting ATRP initiator-functionalized wafers were rinsed with (i) ethanol and (ii) deionized water before drying using a stream of N_2 gas.

Synthesis of 2-(2-nitrophenyl)propoxycarbonyl (3-aminopropyl)triethoxysilane (NPPOC-APTES)

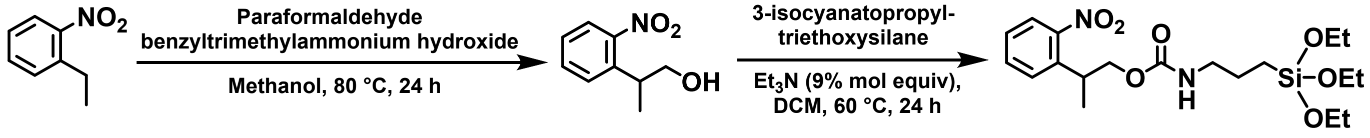

Scheme S1. Two-step synthesis of NPPOC-APTES.

N-(2-(2-Nitrophenyl)propan-1-oxycarbonyl)-3-aminopropyl)triethoxysilane was synthesized synthesized as described previously (Scheme S1).[4] 1-ethyl-2-nitrobenzene (5400 µL, 40.0 mmol) was added to a 40% benzyltrimethylammonium hydroxide (Triton B) solution in methanol (17 g of solution, 40.6 mmol), followed by the addition of paraformaldehyde (1267

mg, 40.5 mmol). The mixture was refluxed at 80 °C for 24 h, evaporated under reduced pressure to a small volume, and adjusted to pH 7 with 1 M aq HCl. The mixture was extracted three times with ethyl acetate (50 mL each), and the organic layers were combined, dried with $MgSO_4$, and evaporated under reduced pressure to give a brown oil. This was purified by flash column chromatography (petroleum spirit 40-60 °C/ethyl acetate, 4:1 2:1) to yield the desired intermediate compound, 2-(2-nitrophenyl)propan-1-ol, as a deep orange oil (3206 mg, 17.69 mmol, 44%). 2-(2-nitrophenyl)propan-1-ol: R_f 0.13 (petroleum spirit 40–60 °C / ethyl acetate 4:1); ν_{max}(neat)/cm^{-1} 3351 (OH), 2972 (alkyl), 2877 (alkyl), 1519 ($ArNO_2$), 1351 ($ArNO_2$), 1035 (Ar), 746 (Ar); ^{1}H NMR (400 MHz, $CDCl_3$) δ 7.77 (dd, *J* = 8.2, 1.4 Hz, 1H, Ar-**H**), 7.60 (td, *J* = 7.6, 1.4 Hz, 1H, Ar-**H**), 7.52 (dd, *J* = 7.9, 1.5 Hz, 1H, Ar-**H**), 7.38 (t, *J* = 7.7 Hz, 1H, Ar-**H**), 3.81 (t, *J* = 5.9 Hz, 2H, C$\mathbf{H_2}$), 3.54 (h, *J* = 6.8 Hz, 1H, C**H**), 1.66 (s(br), 1H, CH_2O**H**), 1.35 (d, *J* = 6.9 Hz, 3H, C$\mathbf{H_3}$); ^{13}C NMR (101 MHz, $CDCl_3$) δ 145.36 (**Ar**), 138.09 (**Ar**), 132.63 (**Ar**), 128.19 (**Ar**), 127.21 (**Ar**), 124.10 (**Ar**), 67.89 (**C**H_2OH), 36.36 (**C**H), 17.53 (**C**H_3); *m/z* (ES+) 202 (100%, $[M + Na]^+$).

Following this, 2-(2-nitrophenyl)propan-1-ol (3206 mg, 17.69 mmol) was dissolved in dichloromethane (15 mL), followed by the addition of 3-(triethoxysilyl)propyl isocyanate (5 mL, 20.2 mmol) and then triethylamine (235 μL, 1.7 mmol). The mixture was refluxed at 60 °C for 24 h. Diethyl ether (20 mL) was added, and the mixture was extracted three times with potassium phosphate buffer (~ 50 mL each). The organic layers were combined, dried with $MgSO_4$, and evaporated under reduced pressure to give a yellow oil. This was purified by flash column chromatography (petroleum spirit 40-60 °C/ethyl acetate, 3:1 2:1) to yield the desired product, NPPOC-APTES, as a yellow oil (1901 mg, 4.44 mmol, 25%). NPPOC-APTES: R_f 0.29 (petroleum spirit 40–60 °C / ethyl acetate 3:1); ^{1}H NMR (400 MHz, $CDCl_3$) δ 7.74 (d, *J* = 1.3 Hz, 1H, Ar-**H**), 7.58 (dd, *J* = 7.6 Hz, 1H, Ar-**H**), 7.48 (d, *J* = 1.4 Hz, 1H, Ar-**H**),

7.38 (dd, J = 7.7 Hz, 1H, Ar-**H**), 4.86 (s(br), 1H, CON**H**CH_2), 4.25 (dd, J = 10.5, 6.1 Hz, 1H, CHC**H₂**O), 4.12 (dd, J = 10.6, 8.2 Hz, 1H, CHC$\mathbf{H_2}$O), 3.82 (q, J = 7.0 Hz, 6H, SiOC$\mathbf{H_2}$), 3.71 (dq, J = 13.3, 6.6 Hz, 1H, CH_3C**H**), 3.14 (q, J = 6.7 Hz, 2H, NHC$\mathbf{H_2}$), 1.59 (m, 2H, CH_2C$\mathbf{H_2}$$CH_2$), 1.36 (d, J = 7.0 Hz, 3H, C$\mathbf{H_3}$CH), 1.23 (t, J = 7.0 Hz, 9H, OCH_2C$\mathbf{H_3}$), 0.61 (t, 2H, C$\mathbf{H_2}$Si); ^{13}C NMR (101 MHz, $CDCl_3$) δ 156.12 (O**C**ONH), 150.95 (**Ar**), 137.53 (**Ar**), 132.56 (**Ar**), 127.96 (**Ar**), 127.28 (**Ar**), 124.01 (**Ar**), 68.53 (CH**C**H_2O), 58.45 (O**C**$H_2$$CH_3$), 43.37 (CONH**C**$H_2$), 33.26 ($CH_3$**C**H), 23.21($CH_2$**C**$H_2$$CH_2$), 18.28 ($OCH_2$**C**$H_3$), 17.46 (**C**$H_3$CH), 7.56 (SiO**C**$H_2$); *m*/*z* (ES+) 451 (100%, $[M + Na]^+$).

Preparation of NPPOC-functionalized surfaces

Silicon wafer substrates and glassware were first cleaned with piranha solution (a mixture of sulfuric acid and hydrogen peroxide in a 7:3 ratio) for 2 h, rinsed thoroughly with deionized water and dried in an oven overnight. To prepare the NPPOC-APTES surfaces, the cleaned silicon wafers were immersed in a 1 mM solution of NPPOC-APTES prepared in toluene for 48 h under continuous nitrogen flow. The samples were then removed, rinsed, sonicated in toluene, 1:1 toluene/ethanol and ethanol for 10 min each to remove any weakly adsorbed material, dried under a stream of nitrogen and annealed in an oven at 120°C for 1 h. Since NPPOC-APTES surfaces are prone to photodegradation, measures were taken to protect the samples from light exposure during the reaction, transport and storage, such as handling the samples under UV-filtered light and wrapping the apparatus and storage vials with aluminum foil.

Preparation of patterned initiator surfaces

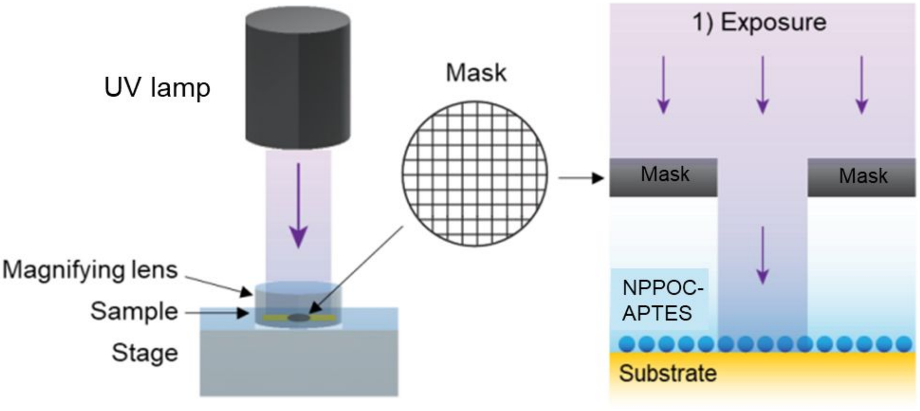

Figure S2. Schematic representation of micropatterning set-up used to prepare NPPOC-APTES-patterned thin films.

A UVP Blak-Ray B-100AP lamp (emission wavelength = 365 nm longwave UV, 100 W) was used to pattern NPPOC-APTES functionalized silicon wafers for emulsion adsorption studies. Micropatterned surfaces were obtained by exposing the NPPOC-APTES functionalized substrates to UV irradiation through a copper mesh grid mask (Agar Scientific, 1500 Mesh TEM Support Grids), held in place by a quartz disk. A dose of 7 J cm^{-2} was used to deprotect the NPPOC-APTES, after which the samples were washed with toluene and dried with a stream of N_2 gas. The freshly prepared samples were transferred into a solution of TEA (0.6 mL, 215.7 mM) and BiBB (0.6 mL, 242.7 mM) in DCM for 1 hour, then rinsed with ethanol and dried with N_2 gas. The patterned PAGEO5MA brushes were obtained by following the polymerization process outlined in *Synthesis and functionalization of PGEO5MA brushes and oxidation to PAGEO5MA*, with a polymerisation time of 1 hour.

Synthesis and functionalization of PGEO5MA brushes and oxidation to PAGEO5MA

PGEO5MA brushes were grown from initiator-functionalized silicon wafers using surface-initiated ARGET ATRP (SI-ARGET ATRP).[2,3] In a typical experiment, the initiator-functionalized substrate was immersed in an aqueous solution comprising GEO5MA (6.48 g,

1.4 M), copper(II) chloride (2.3 mg, 1.4 mM), PMDETA (14.8 mg, 7.1 mM) and deionized water (6.60 g) to afford a final GEO5MA concentration of 45% v/v. Ascorbic acid (30 mg, 14.2 mM) was then added to this aqueous solution, which was stirred for 10 min at 22 °C. The ensuing surface-initiated polymerization was allowed to proceed for 1 h at 22 °C. The wafer was then removed from the reaction solution, rinsed thoroughly with ethanol and deionized water, and dried using a stream of N_2 gas.

Selective oxidation of a *cis*-diol-functional PGEO5MA brush to obtain an aldehyde-functionalized PAGEO5MA brush was conducted using an optimized literature protocol.[2,3] A PGEO5MA brush-decorated wafer was immersed in a 3.0 g dm^{-3} aqueous solution of $NaIO_4$ at 22 °C. After 30 min, the wafer was removed and rinsed thoroughly with ethanol and deionized water, followed by drying using a stream of compressed air.

Adsorption of oil droplets onto a PAGEO5MA brush

The initial 20% w/w Pickering nanomulsion was diluted to 10% w/w with water (adjusted to pH 4, 7 or 10 using HCl or NaOH). A PAGEO5MA-functionalized silicon wafer was immersed into this 10% w/w aqueous nanoemulsion and adsorption was allowed to proceed overnight for at least 16 h at 4 °C. Then the planar substrate was removed from the aqueous dispersion, rinsed with water at the same pH and immediately imaged using either fluorescent microscopy or confocal microscopy. Essentially the same protocol was used for the surface patterned silicon wafers.

Characterization techniques

NMR Spectroscopy

^{1}H and ^{19}F NMR spectra were recorded in either d_4-methanol or d_6-acetone using a Bruker Avance-400 spectrometer operating at 400 MHz (64 scans averaged per spectrum).

Gel Permeation Chromatography (GPC)

An Agilent 1260 Infinity GPC system comprising two Agilent PL-gel 5 μm Mixed-C columns, a guard column, a differential refractive index detector and a UV detector was used to determine the number-average molecular weight (M_n), weight-average molecular weight (M_w), and dispersity (M_w/M_n) for the $PGMA_{52}$ precursor and the final $PGMA_{52}$-$PTFEMA_{50}$ diblock copolymer. HPLC-grade DMF containing 10 mM LiBr was used as the eluent and GPC analysis was performed at 60 °C using a constant flow rate of 1.0 mL min^{-1}. A series of near-monodisperse poly(methyl methacrylate) calibration standards with M_p values ranging from 800 to 2 200 000 g mol^{-1} were used to calculate molecular weights and dispersities. Both samples were diluted to 1.0% w/w using the GPC eluent and chromatograms were analyzed using Agilent GPC/SEC software.

Dynamic Light Scattering (DLS)

Intensity-average size distributions and z-average hydrodynamic diameters, D_z, were obtained at a scattering angle of 173° using a Malvern Zetasizer Nano ZS instrument. A 0.1% w/w aqueous dispersion of $PGMA_{52}$–$PTFEMA_{50}$ nanoparticles and the corresponding Pickering nanoemulsion were analyzed using disposable plastic cuvettes at 20 °C. In each case, data were averaged over three consecutive runs.

Transmission Electron Microscopy (TEM)

Copper/palladium TEM grids (Agar Scientific, U.K.) were coated with a thin film of amorphous carbon. If required, grids were subjected to a plasma glow discharge for 30 s to produce a hydrophilic surface. One droplet of an aqueous dispersion of nanoparticles (0.2% w/w, 10 μL) or a nanoemulsion (0.5% v/v, 10 μL) was placed on a grid for 1 min, after which any remaining solution was removed by blotting with filter paper. Subsequently, an aqueous droplet of uranyl formate (0.75% w/w, 10 μL) was placed on the sample-loaded grid for 20 s, and the excess stain was removed by blotting. Each grid was carefully dried using a vacuum hose. Images

were recorded using an FEI Tecnai Spirit microscope operating at 80 kV and equipped with a Gatan 1kMS600CW CCD camera.

Analytical Centrifugation (LUMiSizer)

The droplet size distribution of the Pickering nanoemulsion was analyzed using a LUMiSizer analytical photocentrifuge (LUM GmbH, Berlin, Germany) at 20 °C. Measurements were conducted on 1.0% v/v Pickering nanoemulsions using 2 mm path length polyamide cells at 500, 1000, 2000 rpm for 200 profiles each as well as 4000 rpm for 400 profiles (with 10 s between each profile). The LUMiSizer instrument uses space- and time-resolved extinction profiles (STEP) technology to measure the intensity of transmitted near-infrared light as a function of time and position over the entire cell length. The gradual progression of transmission profiles contained information on the rate of creaming, which enables calculation of the droplet size distribution. The droplet density is an essential input parameter for analytical centrifugation measurements. This parameter was taken to be 0.81 g cm^{-3}, which is the density of squalane. This approximation ignores any contribution from the $PGMA_{52}$–$PTFEMA_{50}$ nanoparticles adsorbed at the oil droplet surface.

Atomic force microscopy

AFM imaging was performed using a Bruker Nanoscope VIII Multimode Atomic Force Microscope equipped with a ‘J’ scanner. Silicon cantilevers (OTESTPA-R4, Bruker, UK) with a nominal spring constant of 26 N·$m^{=1}$ and a tip radius of 7 nm were used for tapping mode imaging. AFM images were recorded at 22 °C for patterned PGEO5MA brushes in air.

Confocal microscopy

The initial 10% w/w Pickering nanomulsion was diluted to 1.0% w/w with water (adjusted to pH 4, 7 or 10 using HCl or NaOH). A PAGEO5MA-functionalized silicon wafer was immersed into this 1.0% w/w aqueous nanoemulsion and adsorption was allowed to proceed overnight

for at least 16 h at 4 °C. Subsequently, the planar substrate was removed from the aqueous dispersion, rinsed, immersed in water at the same pH, and immediately imaged. Confocal microscopy imaging was performed using a Zeiss LSM 800 confocal microscope equipped with a Plan-Apochromat 40x/1.3 Oil DIC M27 objective. The excitation wavelength was set to 559 nm and the emission maximum was collected at 636 nm.

Ellipsometric characterization of the dry brush thickness

Dry brush thicknesses were determined via spectroscopic ellipsometry using a J. A. Woollam M2000 V ellipsometer at a fixed angle of incidence of 75° normal to the sample surface. A wavelength range of 370–1000 nm was used to obtain two ellipsometric parameters (Ψ and Δ). Measurements were performed in air at 20 °C. Data analysis and modeling were performed using Woollam CompleteEase software, which fits the Ψ and Δ values. The interfacial structure was assumed to be composed of polymer and silica slabs, each describing their respective refractive index and thickness, plus a silicon backing layer. The polymer layer was treated as a Cauchy layer with the following parameters: A_n = 1.4615, B_n = 0.00514 μm^{-2}, and C_n = 0 μm^{-4}. The silica layer thickness was allowed to vary between 1 and 3 nm, with a typical value being ~1.5 nm.

Quartz crystal microbalance (QCM) measurements

QCM sensors coated with a 50 nm silica overlayer (QSX 303, ~5 MHz fundamental frequency) were purchased from Q-Sense (Sweden). Each sensor was cleaned according to the manufacturer's instructions. This protocol involved (i) UV/ozone treatment for 15 min (Bioforce UV/Ozone cleaner, ~9 mW cm^{-2}, λ = 254 nm), (ii) exposure to 2% w/w sodium dodecylsulfate solution for 30 min, (iii) copious rinsing with deionized water and drying under N_2, and (iv) a final UV/ozone treatment for 15 min. The resulting substrates were functionalized with initiator groups prior to surface-initiated polymerization to produce brush-coated substrates.

All QCM experiments were performed at 25°C using an openQCM NEXT instrument (Novatech Srl., Italy) equipped with a temperature-controlled cell connected to a Masterflex Digital Miniflex peristaltic pump (Cole-Parmer Instrument Company, UK). Initially, a rapid flow of ethanol (2.0 ml min^{-1}) was used to wet the sensor surface. Once all bubbles had been removed, pH adjusted water was flowed through the cell until the sensor frequency exhibited a drift of less than 1 Hz min^{-1}. This typically occurred within 1 h. Once a stable signal was obtained, 1.0% w/w Pickering nanoemulsion was passed through the cell at a flow rate of 0.1 mL min^{-1} (minimum flow volume = 3.0 mL). Once any signal drift had abated, phosphate buffer solution was passed through the cell at the same flow rate. The adsorbed amount was calculated using the Sauerbrey equation, which relates the change in frequency, Δf, to the change in adsorbed mass per unit area, *m*.

$$m = C \times \frac{\Delta f}{n}$$

where *C* is a sensitivity constant −0.177 mg m^{-2} Hz^{-1}, Δf is the change in resonant frequency (Hz), and *n* is the overtone number. The third harmonic (n = 3) was used to calculate the adsorbed amount (mg m^{-2}) to avoid experimental artifacts associated with the fundamental harmonic. The Sauerbrey equation can be applied when the $\Delta D_n/\Delta f_n$ ratio is less than 0.1 × 10^{-7} Hz^{-1} and minimal overtone dependence on Δf_n.[5,6]

References

(1) Brotherton, E. E.; Jesson, C. P.; Warren, N. J.; Smallridge, M. J.; Armes, S. P. New Aldehyde-Functional Methacrylic Water-Soluble Polymers. *Angewandte Chemie International Edition* **2021**, *60* (21), 12032–12037. https://doi.org/10.1002/anie.202015298.

(2) Brotherton, E. E.; Johnson, E. C.; Smallridge, M. J.; Hammond, D. B.; Leggett, G. J.; Armes, S. P. Hydrophilic Aldehyde-Functional Polymer Brushes: Synthesis, Characterization, and Potential Bioapplications. *Macromolecules* **2023**, *56* (5), 2070–2080. https://doi.org/10.1021/acs.macromol.2c02471.

(3) Johnson, E. C.; Varlas, S.; Norvilaite, O.; Neal, T. J.; Brotherton, E. E.; Sanderson, G.; Leggett, G. J.; Armes, S. P. Adsorption of Aldehyde-Functional Diblock Copolymer Spheres onto Surface-Grafted Polymer Brushes via Dynamic Covalent Chemistry Enables Friction Modification. *Chemistry of Materials* **2023**, *35* (15), 6109–6122. https://doi.org/10.1021/acs.chemmater.3c01227.

(4) Alang Ahmad, S. A.; Wong, L. S.; ul-Haq, E.; Hobbs, J. K.; Leggett, G. J.; Micklefield, J. Micrometer- and Nanometer-Scale Photopatterning Using 2-Nitrophenylpropyloxycarbonyl-Protected Aminosiloxane Monolayers. *J Am Chem Soc* **2009**, *131* (4), 1513–1522. https://doi.org/10.1021/ja807612y.

(5) Reviakine, I.; Johannsmann, D.; Richter, R. P. Hearing What You Cannot See and Visualizing What You Hear: Interpreting Quartz Crystal Microbalance Data from Solvated Interfaces. *Anal Chem* **2011**, *83* (23), 8838–8848. https://doi.org/10.1021/ac201778h.

(6) Cho, N.-J.; Frank, C. W.; Kasemo, B.; Höök, F. Quartz Crystal Microbalance with Dissipation Monitoring of Supported Lipid Bilayers on Various Substrates. *Nat Protoc* **2010**, *5* (6), 1096–1106. https://doi.org/10.1038/nprot.2010.65.
